# Can We Predict the Motor Performance of Patients With Parkinson's Disease Based on Their Symptomatology?

**DOI:** 10.3389/fbioe.2020.00189

**Published:** 2020-03-24

**Authors:** Karina Lebel, Christian Duval, Etienne Goubault, Sarah Bogard, Pierre. J. Blanchet

**Affiliations:** ^1^Département de Génie électrique et de Génie Informatique, Faculté de Génie, Université de Sherbrooke, Sherbrooke, QC, Canada; ^2^Centre de Recherche sur le Vieillissement, Sherbrooke, QC, Canada; ^3^Laboratoire de Simulation et Modélisation du Mouvement, École de Kinésiologie et des Sciences de l'activité physique, Université de Montréal, Montreal, QC, Canada; ^4^Centre de Recherche Institut Universitaire de Gériatrie de Montréal, Montreal, QC, Canada; ^5^Faculté de Médecine Dentaire, Université de Montréal, Montreal, QC, Canada; ^6^Centre Hospitalier de l'Université de Montréal (C.H.U. Montreal), Montreal, QC, Canada

**Keywords:** mobility, motor impact, Parkinson, clustering, K-means

## Abstract

**Introduction:** Parkinson's disease hinders the ability of a person to perform daily activities. However, the varying impact of specific symptoms and their interactions on a person's motor repertoire is not understood. The current study investigates the possibility to predict global motor disabilities based on the patient symptomatology and medication.

**Methods:** A cohort of 115 patients diagnosed with Parkinson's disease (mean age = 67.0 ± 8.7 years old) participated in the study. Participants performed different tasks, including the Timed-Up & Go, eating soup and the Purdue Pegboard test. Performance on these tasks was judged using timing, number of errors committed, and count achieved. K-means method was used to cluster the overall performance and create different motor performance groups. Symptomatology was objectively assessed for each participant from a combination of wearable inertial sensors (bradykinesia, tremor, dyskinesia) and clinical assessment (rigidity, postural instability). A multinomial regression model was derived to predict the performance cluster membership based on the patients' symptomatology, socio-demographics information and medication.

**Results:** Clustering exposed four distinct performance groups: *normal* behavior, slightly affected in fine motor tasks, affected only in TUG, and affected in all areas. The statistical model revealed that low to moderate level of dyskinesia increased the likelihood of being in the *normal* group. A rise in postural instability and rest tremor increase the chance to be affected in TUG. Finally, LEDD did not help distinguishing between groups, but the presence of Amantadine as part of the medication regimen appears to decrease the likelihood of being part of the groups affected in TUG.

**Conclusion:** The approach allowed to demonstrate the potential of using clinical symptoms to predict the impact of Parkinson's disease on a person's mobility performance.

## Introduction

Parkinson's disease (PD) is a neurodegenerative disease characterized by both motor and non-motor symptoms, including tremor, postural instability, muscle rigidity, and bradykinesia or akinesia (Sveinbjornsdottir, 2016). These symptoms affect the ability of patients to perform activities of daily living (ADL) to a varying extent. There is currently no cure for PD, and symptoms are chiefly managed with medication. While the treatment goal is to maximize the person's ability to perform everyday tasks, the impact of each symptom on ADL, and most importantly, of the combination of symptoms, is not well-understood. Past studies have tried to identify different phenotypes in PD to help with this issue, and to guide diagnosis, prognosis, and treatment (Eisinger et al., 2019). These studies identified a tremor dominant subtype and a postural instability gait disorder group (Foltynie et al., 2002). Some studies also recognize an indeterminate subtype to PD, while others propose further sub-groups such as axial dominant, appendicular dominant, and rigidity dominant (Eisinger et al., 2017). In other words, classical approaches for PD phenotyping is mainly based on an *a priori* hypothesis of the importance of the dominant motor symptom on the patient's ability to perform ADL. Yet, patients with PD are often affected by more than one symptom. Combination of symptoms may exacerbate mobility issues or limit the efficacy of compensatory strategies. Furthermore, recent studies have outlined the impact of non-motor symptoms on the patients' ability to perform various ADL (Berganzo et al., 2016). The heterogeneity of the clinical profiles associated with PD therefore result in an unclear relationship between the traditional PD subgroups and the patients' proficiency in ADL. Thus, it appears desirable to revise this classification to allow a better correspondence with the treatment goal. One way to do so is to redirect the sub-typing approach toward an understanding of the functional impact of a patient's symptomatology on its global motor repertoire. Functional impact of a disease is traditionally assessed using questionnaires (Shulman et al., 2016). However, self-reported questionnaire are inclined to over or under-estimation of the patient's ability to perform activities and may suffer from flooring effect, as recently demonstrated by Regnault et al. (2019) for early Parkinson's disease. In an attempt to shed light on this type of issue, our lab has been working on developing methodologies to assess and objectively quantify symptoms and motor skills performance to better understand the relationship between PD symptoms and motor skills performance. We herein set to explore the capacity to assess the impact of the different symptoms on the motor skills repertoire in a global fashion. Specifically, this study aimed at: (1) exploring motor skill performance profiles in patients with PD; (2) identifying the factors (in our case symptoms) influencing the affiliation with a specific motor performance profile; and (3) verifying the possibility to create a model allowing to predict the motor performance profile based on the symptomatology.

## Methods

### Participants

**Figure 1:**
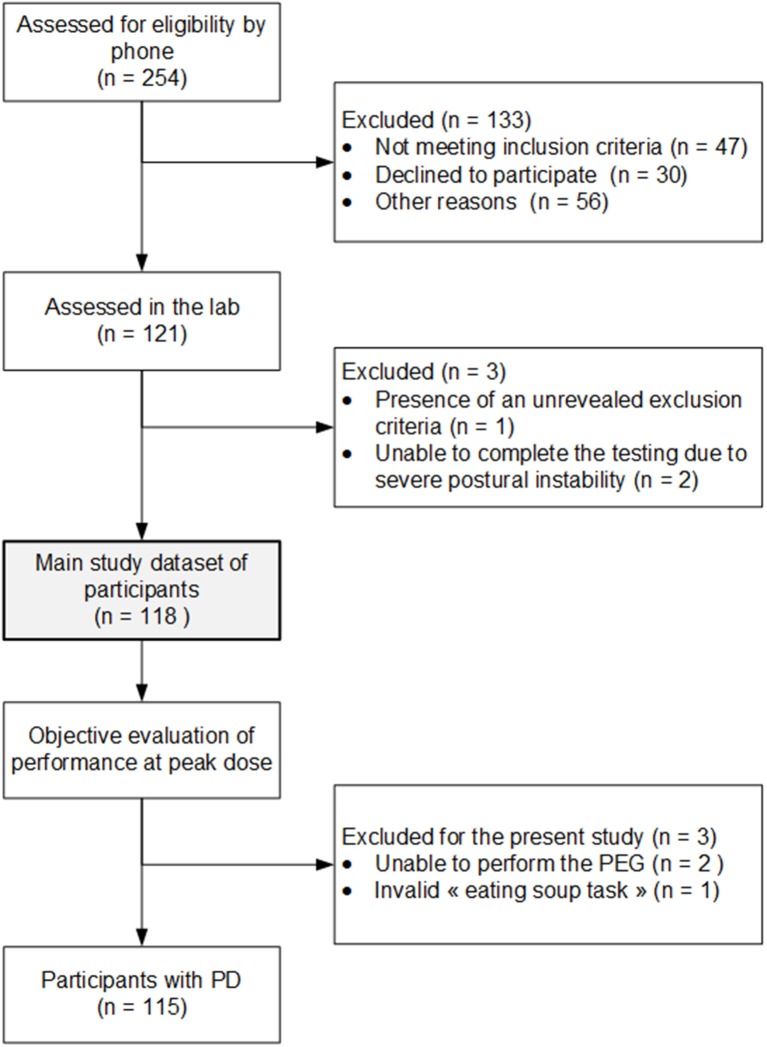
Study inclusion flowchart.

Data were extracted from a cohort of 121 patients diagnosed with PD. These participants were recruited in collaboration with the Quebec Parkinson Network and the Movement Disorders Clinic of the University of Calgary. Inclusion criteria consisted of a valid PD diagnosis given by a neurologist based on the UK Parkinson's Disease Society Brain Bank clinical diagnosis criteria (Hughes et al., 1992). Patients requiring assistance to walk, having an orthopedic condition that could hinder the performance of the tasks, as well as patients with a psychosis, were all excluded from the study. Previous publications using the data bank focused on the concomitant presence of cardinal symptoms of PD with dyskinesia (Goubault et al., 2018), as well as the influence of dyskinesia on motor performance (Goubault et al., 2019). For the present study, six additional participants were excluded as detailed in Figure 1. As a result, a sample of 115 patients, described in Table 1, was considered for the present study.

**Table 1:** Study participants description.

	**Healthy elderly (*n* = 69)**	**Parkinson's disease patients (*n* = 115)**	***p***
**GENERAL**			
Gender (% male)	56.5%	58.3%	*P* = 0.8779
Age (yr)	68.1 ± 7.7	67.0 ± 8.7	*P* = 0.4246
Height (m)	1.67 ± 0.09	1.69 ± 0.10	*P* = 0.4804
Weight (Kg)	71.3 ± 14.5	69.9 ± 13.1	*P* = 0.6188
BMI	25.3 ± 3.9	24.5 ± 4.0	*P* = 0.1928
MMSE (/30)	28.6 ± 1.5	27.3 ± 2.5	*P* < 0.001
**DISEASE INFO**			
H&Y	–	1: 22.6% 3: 15.7% 2: 53.9% 4: 7.8%	–
UPDRS gait	–	1.1 ± 0.9	–
UPDRS freezing of gait	–	0.3 ± 0.8	–
UPDRS postural stability	–	1.1 ± 1.0	–
UPDRS posture	–	0.8 ± 1.0	–
UPDRS global spontaneity of movement	–	1.1 ± 1.0	–
UPDRS Postural tremor	–	0.5 ± 0.9	–
UPDRS rest tremor	–	0.2 ± 0.6	–
UPDRS rigidity	–	*Arms:* 0.7 ± 0.7	–
		*Legs:* 1.1 ± 0.7	–
Years since diagnosis	–	10.5 ± 5.8	–
**MEDICATION**			
LEDD	–	1029.1 ± 509.2^**^	–
Levodopa (%)	–	100^*^	–
Agonist (%)	–	32.7^*^	–
Amantadine (%)	–	39.8^*^	–
COMT or MAOB (%)	–	49.6^*^	–

*BMI, Body Mass Index; MMSE, Mini-Mental State Exam; H&Y, Hoehn and Yahr scale; UPDRS, Unified Parkinson's Disease Rating Scale; LEDD, Levodopa Equivalent Daily Dose*.

* Missing medication profile for 2 participants;

** *Missing info for 6 participants*.

A second group of participants composed of 69 age and gender-matched community-dwelling elderly (43.5% female, age = 68.1 ± 7.7 years old, BMI = 25.3 ± 3.9) was also recruited through the Center de Recherche de l'Institut universitaire de gériatrie de Montréal (CRIUGM) to provide control data. The study protocol was approved by both the CRIUGM and the Conjoint Health Research ethics boards, and all participants provided written informed consent.

### Experimental Protocol

The experimental protocol has been described in detail previously (Goubault et al., 2018, 2019). In brief, participants were tested on their regular medication and equipped with an inertial suit containing 17 sensors (IGS-180, Synertial Ltd, UK), allowing the capture of the entire body kinematics. Each sensor is composed of a 3-axis accelerometer, measuring linear acceleration, a 3-axis gyroscope, assessing angular velocity, and a 3-axis magnetometer. Upon arrival to the lab, participants took their medication and were asked to fill-up a socio-demographic questionnaire as well as cognitive and quality of life questionnaires. The study's objective data acquisition process was then divided into two blocks of ADL, nested between blocks of symptoms evaluation. The chosen ADL included a variety of activities corresponding to a wide range in velocity and amplitude of motion (upper and/or lower limbs), in order to represent the complete motor repertoire. Tasks were selected from a variety of ADL and instrumented ADL scales (Klein and Bell, 1982; Fahn et al., 1987; Lozano et al., 1995; Boraud et al., 2001; Health Canada Interdepartmental Committee on Aging Seniors Issues, 2001; Krystkowiak and Defebvre, 2002; Guttman et al., 2003; Health Canada/Parkinson Society Canada, 2003; Goetz et al., 2008; Colosimo et al., 2010; Carignan et al., 2011). Chosen tasks included eating soup, cutting and eating a piece of apple, taking pills, drinking a glass of water, counting money, reading a book out loud, reaching to grab an object on the ground, rising from a chair, walking, turning, sitting down (Timed-Up and Go, TUG), and the Purdue Pegboard task. Participants were cued to initiate the task when a light, positioned in front of them, turned on. For this specific study, a subset of tasks was considered in order to limit the degrees of freedom in the analysis. The selected tasks included the TUG, eating soup (ES), and the Purdue Pegboard test (PB). While ES involves short range, slow speed movements, PB requires short range and fast motion, while the TUG relates to long range, medium speed global motion. For ES, participants sat down on a bench with both hands flat on the table. Once cued by the light, participants were instructed to take the spoon positioned on the table using their dominant hand, take four spoons of water at their preferred pace to reproduce true living conditions, return the spoon to the table, and position their hands back on the table. The time required to performed the task corresponds to the time elapsed between the light stimuli and the time the hands are placed back on the table. For PB, a board with two parallel rows composed of 25 holes each was placed in front of the participant. Upon signal, participants were instructed to insert as many pins in the holes as possible in 30 s, using both hands alternately. The TUG was initiated with the participant sitting on a bench. Upon signal (i.e., light), the participant was asked to rise from the bench without any help if possible (i.e., no hands), walk for 3 m at their preferred pace, and return to their initial sitting position. Performance was assessed using the time required to perform the task (ES, TUG), the count achieved (PB), and the number of errors committed (ES: dropping water, dropping the spoon; TUG: needing assistance, using hands to rise/sit; PB: dropping pins).

The symptoms assessment blocks consisted of a mixture of clinical evaluation and objective assessment of the symptomatology: postural instability was assessed using the pull-back test (Unified Parkinson's Disease Rating Scale item 3.12), rigidity was evaluated manually for each limb (item #3.3), bradykinesia was appraised objectively using a rapid alternating task, while tremor, drug-induced dyskinesia (DID) and freezing of gait (FoG) were all assessed objectively during appropriate tasks using inertial data (Goubault et al., 2018). Briefly, tremor was assessed using the signal captured by the gyroscopes positioned on the hands. These signals were band-pass filtered between 3 and 7.5 Hz to isolate the tremor frequency range. A power density spectrum was then used to identify the signal dominant frequency, as well as its dispersion. Tremor was detected when dispersion was below 2 Hz, in which case the corresponding tremor value was fixed to the dispersion bandwidth. DID was assessed during the tasks, using signals from the sensors not directly involved in the specific task. Signals from the gyroscopes were again band-pass filtered, this time between 0.5 and 4 Hz. The energy of the resulting signal was then computed, per segment. The average energy among the different segments considered corresponds to the DID value attributed for the task. Freezing of gait was assessed during the walking portion of the TUG. The process uses the ratio of the power of the signal within the walking bandwidth to the power located within the freezing bandwidth to identify freezing events.

### Performance Clusters Identification

A clustering approach was used to explore the presence of motor skills performance profiles within a group of patients medicated for PD. This method allows the groups to emerge directly from the data without bias (Rui and Wunsch, 2005). In this specific case, performance clusters were based on five metrics extracted from three selected tasks: TUG time, TUG errors, Eating soup time, Eating soup errors, and Pegboard number of pins. To ensure all metrics have a similar influence during the clustering process, timing features as well as the Pegboard pins count were first normalized based on the control group performance data. Extreme values, defined as values outside the ±4 Z-score, were also set to the closest valid limit.

Clustering was performed using the K-means method. In brief, this approach uses an iterative process to minimize the sum of the distances between each point and its cluster's centroid, while maximizing the difference between the clusters (Rui and Wunsch, 2005). This method, however, requires the user to specify the desired number of clusters. We defined the ideal number of clusters as a trade-off between the sum of the Euclidean distance between each point and its cluster's centroid and the resulting number of very small clusters, herein defined as groups composed of fewer than 10 participants. In other words, the clustering process was performed using a varying number of clusters, from 1 to 115 (the number of participants), and the quality of the resulting clusters was evaluated based on both the distance cost and the resulting number of small clusters, to identify the optimal number of clusters. The ability of the clusters to differentiate performance was then evaluated using a Kruskall-Wallis ANOVA test. The clustering and validation processes were performed in Matlab Release 2018a (The MathWorks, Massachusetts, United States).

### Performance Profiles Features Identification & Membership Prediction

The second objective of this study consists in analyzing which features, amongst the motor and the non-motor symptoms as well as the participants' characteristics, explain the affiliation to a specific motor performance profile or cluster. To do so, symptomatology was first normalized based on the control group data acquired. Then, the sample was divided into a training and a validation datasets (80–20%). Using the training dataset, univariate multinomial regression analysis was performed on each variable, that is age, gender, BMI, years living with PD, Mini-Mental State Exam (MMSE), symptomatology (dyskinesia, bradykinesia, rest tremor, postural tremor, kinetic tremor, rigidity, postural instability, freezing of gait), and medication regimen [Levodopa Equivalent Daily Dose (LEDD), Levodopa, Agonist, Amantadine, COMT or MAOB]. All variables with a marginal significance (i.e., *p*-value) smaller or equal to 0.2 were identified as potentially explicative variables (PEV) for a specific cluster membership. A multivariate multinomial regression analysis was then performed using these PEV. The model was designed using 80% of the sample; and verified with the remaining 20%. The accuracy of the proposed model was then evaluated based on a contingency table. All statistical analyses were performed using SPSS v23 (IBM Corp., Armonk, NY).

## Results

### Clusters Identification Results

**Figure 2:**
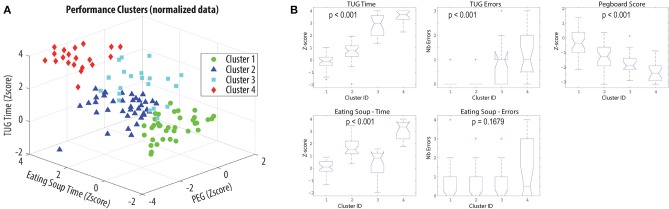
Performance clusters identification. **(A)** Visual representation of the clusters based on a subset of 3 factors. **(B)** Boxplots of the distribution of the different performance factors within the clusters. The *p*-values correspond to the result of the Kruskall-Wallis ANOVA test.

Four clusters of performance were identified (Figure 2A) and confirmed by statistical analyses. Only the number of errors made while eating soup was not shown to be a discriminative factor (Figure 2B). As detailed in Table 2, Cluster 1 is composed of participants who performed within normal for all tasks and parameters. Cluster 2 corresponds to participants slightly affected in fine motor tasks. Cluster 3 is made of participants mainly affected during the TUG, while the last cluster is composed of participants affected in all activities.

**Table 2:** Clusters performance details.

	**Cluster ID**				***p***
	**1**	**2**	**3**	**4**	
**TUG time (s)** *Median [Q1,Q3]*	13.0 [12.3, 13.6] $\begin{array}{c} \text{Not}\;\text{affected} \end{array}$	14.6 [13.7, 15.5] $\begin{array}{c} \text{Not}\;\text{affected} \end{array}$	20.2 [17.5, 22.2] $\begin{array}{c} \text{Affected} \end{array}$	22.4 [21.2, 25.0] $\begin{array}{c} \text{Affected} \end{array}$	*p* < 0.001
**TUG err** *Median [Q1,Q3]*	0 [0, 0] $\begin{array}{c} \text{Not}\;\text{affected} \end{array}$	0 [0, 0] $\begin{array}{c} \text{Not}\;\text{affected} \end{array}$	1.0 [0, 1.0] $\begin{array}{c} \text{Affected} \end{array}$	1.0 [0.5, 2.0] $\begin{array}{c} \text{Affected} \end{array}$	*p* < 0.001
**Pegboard #pins** *Median [Q1,Q3]*	15.0 [12.5, 18.0] $\begin{array}{c} \text{Not}\;\text{affected} \end{array}$	11.5 [9.0, 14.0] $\begin{array}{c} \text{Slightly}\;\text{affected} \end{array}$	9.0 [8.0, 11.0] $\begin{array}{c} \text{Slightly}\;\text{affected} \end{array}$	7.0 [5.0, 9.0] $\begin{array}{c} \text{Affected} \end{array}$	*p* < 0.001
**Eating Soup time (s)** *Median [Q1,Q3]*	18.9 [17.9, 20.4] $\begin{array}{c} \text{Not}\;\text{affected} \end{array}$	23.9 [22.6, 26.8] $\begin{array}{c} \text{Slightly}\;\text{affected} \end{array}$	21.3 [17.5, 22.8] $\begin{array}{c} \text{Not}\;\text{affected} \end{array}$	32.3 [27.6, 34.3] $\begin{array}{c} \text{Affected} \end{array}$	*p* < 0.001
**Eating Soup Errors** *Median [Q1,Q3]*	0 [0, 1]	0 [0, 1]	0 [0, 1]	0.5 [0, 3]	*p* = 0.1679

### Performance Features Identification Results

Cluster membership was attributed to each participant, following the process described in section Performance Profiles Features Identification & Membership Prediction. The resulting portrait of the patients' symptomatology profiles, per cluster, is reported in Table 3.

**Table 3:** Patients symptomatology portrait per performance cluster.

	**Cluster ID**			
	**1** **(*n* = 36)**	**2** **(*n* = 38)**	**3** **(*n* = 21)**	**4** **(*n* = 20)**
**GENERAL**				
Gender (% male)	52.8%	68.4%	61.9%	45.0%
Age (yr)	64.0 ± 8.7	66.5 ± 8.4	70.0 ± 8.7	70.2 ± 7.5
BMI	23.9 ± 3.3	24.0 ± 3.8	24.9 ± 3.4	26.0 ± 5.5
MMSE (/30)	28.2 ± 1.5	27.9 ± 2.0	26.5 ± 2.6	25.3 ± 3.4
**DISEASE INFO**				
H&Y	1: 41.7%	1: 23.7%	1: 9.5%	1: 0.0%
	2: 55.6%	2: 68.4%	2: 33.3%	2: 45.0%
	3: 2.8%	3: 7.9%	3: 47.6%	3: 20.0%
	4: 0.0%	4: 0.0%	4: 9.5%	4: 35.0%
Years since diagnosis	8.8 ± 3.7	10.6 ± 5.9	11.8 ± 7.7	12.2 ± 6.4
**MEDICATION**				
LEDD	994.9 ± 450.8	866.0 ± 448.1	1378.8 ± 567.4	1051.5 ± 523.7
Levodopa (%)	100%	100%	100%	100%
Agonist (%)	38.9%	35.1%	35.0%	15.0%
Amantadine (%)	50.0%	35.1%	15.0%	55.0%
COMT or MAOB(%)	47.2%	59.5%	40.0%	45.0%
**SYMPTOMS**				
Dyskinesia (normalized value)	1.1 ± 1.6 [−1.7, 4.2]	−0.5 ± 1.4 [−2.7, 2.5]	0.6 ± 2.1 [−3.2, 3.2]	−0.8 ± 2.2 [−4.1, 4.1]
Bradykinesia (normalized value)	−1.1 ± 1.1 [−3.9, 0.7]	−1.9 ± 1.8 [−6.0, 1.1]	−1.7 ± 1.6 [−4.6, 1.1]	−3.6 ± 1.5 [−6.0, −0.9]
Rest tremor (normalized value)	0.3 ± 4.1 [−6.0, 6.0]	0.7 ± 3.9 [−6.0, 6.0]	1.1 ± 4.3 [−6.0, 6.0]	2.8 ± 3.2 [−2.3, 6.0]
Postural tremor (normalized value)	[1.6, 6.0]	[1.6, 6.0]	[1.6, 6.0]	[1.6, 6.0]
Kinetic tremor (normalized value)	0.5 ± 1.8 [−2.8, 6.4]	−0.1 ± 1.8 [−3.0, 4.9]	1.6 ± 2.1 [−2.1, 5.5]	1.3 ± 2.3 [−3.5, 6.0]
Postural instability	0.7 ± 0.7 [0.0, 2.5]	0.7 ± 0.7 [0.0, 1.0]	2.1 ± 1.2 [0.0, 4.0]	1.9 ± 1.0 [1.0, 4.0]
Freezing (%)	0.0 ± 0.0 [0.0, 0.0]	0.2 ± 1.2 [0.0, 2.0]	5.0 ± 15.7 [0.0, 71.6]	3.8 ± 9.0 [0.0, 36.6]
Rigidity	0.8 ± 0.6 [0, 2.5]	0.9 ± 0.6 [0, 2.0]	0.8 ± 0.8 [0, 2.5]	1.3 ± 0.6 [0.2, 2.5]

Univariate multinomial analysis performed on this set of data allowed to identify 10 potentially explanatory variables: age (*p* = 0.134), MMSE (*p* = 0.200), dyskinesia (*p* < 0.001), bradykinesia (*p* < 0.001), rest tremor (*p* = 0.024), kinetic tremor (*p* = 0.010), rigidity (*p* = 0.010), postural instability (*p* < 0.001), freezing of gait (*p* < 0.001), and the presence of Amantadine in the medication regimen (*p* = 0.180). Including all these potentially explanatory variables into a single multinomial regression allowed to derive a significant model (χ^2^ = 140.628, *p* < 0.001) with a good representativeness (Nagelkerke pseudo R^2^ = 0.839). This global model identified postural instability (*p* < 0.001), dyskinesia (*p* = 0.024), bradykinesia (*p* = 0.022), rigidity (*p* = 0.026), freezing of gait (*p* = 0.040), as well as Amantadine (*p* = 0.003) as the main significant variables, while cognitive impairment (*p* = 0.064) and rest tremor (*p* = 0.086) significantly discriminates between sub-groups 3 and 1 despite being globally significant.

Detailed analysis of the model revealed that:

- an increase in postural instability increases the chance to be part of cluster 3 or 4, relative to cluster 1 or 2 (p_3rel1_ = 0.001, OR_3rel1_ = 9.323 [2.430, 35.773]; p_3rel2_ = 0.001, OR_3rel2_ = 6.785 [2.107, 21.851]; p_4rel1_ = 0.009, OR_4rel1_ = 6.268 [1.574, 24.957]; p_4rel2_ = 0.012, OR_4rel2_ = 4.561 [1.399, 14.868]);
- an increase of one standard deviation in dyskinesia level increases the chance to be in cluster 1 compared to cluster 2 or 3 (p_1rel2_ = 0.014, OR_1rel2_: 2.12 [1.17, 3.86]; p_1rel3_ = 0.023, OR_1rel3_ = 2.92 [1.16, 7.30]);
- an increase of one standard deviation in bradykinesia level increases the likelihood of being in cluster 4 relative to cluster 3 (*p* = 0.025, OR = 6.06 [1.26, 29.41]);
- an increase in rigidity increases the chance to be in cluster 4 relative to cluster 1 (*p* = 0.025; OR = 34.17 [1.54, 757.05]) and cluster 2 (*p* = 0.036, OR = 24.97 [1.23, 506.53]);
- the presence of Amantadine in the medication regimen appears to decrease the risk of being in cluster 3, when compared to cluster 1 or 2 (p_3rel1_ = 0.025, OR_3rel1_ = 3.22E-4 [2.92E-7, 0.356]; p_3rel2_ = 0.044, OR_3rel2_ = 0.001 [7.30E-7, 0.825]).

This model allowed to classify the participants within their respective cluster of performance with an accuracy of 76%, as illustrated in the contingency table (Table 4).

**Table 4:** Contingency table.

		**Predicted cluster**				
		**1**	**2**	**3**	**4**	**% Correct**
Observed cluster	1	27	7	1	1	75.0%
	2	9	26	0	2	70.3%
	3	1	2	16	1	80.0%
	4	1	2	0	17	85.0%
	Overall percentage	33.6%	32.7%	15.0%	18.6%	76.1%

## Discussion

This study first aimed at investigating the presence of motor skills performance profiles in patients medicated for PD. Using a clustering approach, four different profiles emerged from the data. Analyzing the variation in metrics within each cluster revealed that cluster 1 is composed of participants who are not affected in the motor tasks assessed under medication. Cluster 2 participants are affected only slightly in fine motor tasks. Cluster 3 participants are mainly affected in mobility tasks, while cluster 4 involves participants affected in all areas. These clusters were shown to be statistically different for four performance metrics out of five, demonstrating the potential of the method. This approach offers an innovative view for PD classification, focussed on the global impact of the disease on the patient's motor repertoire as opposed to a more classical dominant symptom classification (Foltynie et al., 2002; Eisinger et al., 2017, 2019; Erro et al., 2019). To our knowledge, this study is the first to address the phenotype problematic from this point of view. Direct comparison between the two classification approaches would be worth investigating. Nevertheless, it is clear from the description of the symptomatology profile per cluster that symptoms coexist within the clusters. This observation supports a global approach of symptomatology characterization for motor performance prediction.

Although the clusters identified are statistically significant and appear to hold a clinical meaning, it shall be noted that the clustering method could be further refined. Indeed, the K-mean method requires the user to determine in advance the number of desired clusters. In order to remain as objective as possible, we first investigated different potential avenues for clusters quantity identification, such as the use of the silhouette validity index and the Calinski-Harabasz index (Arbelaitz et al., 2013). However, Hennig (2015) exposed an interesting way of looking at true clusters based on the direct aim pursued by the clustering process. Indeed, the idea for true or ideal clusters may vary depending on the situation. In the current study, we know that the optimal number of clusters represents different mobility profiles, however somehow subtle these differences may be. As such, the dissimilarity between clusters criterion may not be obvious, and thus, the classic validity indexes may not be optimal. As such, we identified the ideal number of clusters as a trade-off between the within clusters similarity and the number of small clusters created. The pragmatic approach was appropriate for the current study, but may need to be revisited in other cases.

The second part of the study aimed at exploring potential factors influencing the affiliation of a participant to a specific cluster of performance in the “ON” medicated state. It was shown that postural instability, dyskinesia, bradykinesia, rigidity, freezing of gait, and Amantadine all play a significant role in the classification process. Consistent with the literature, postural instability and freezing of gait discriminated patients with disabled mobility (Muslimovic et al., 2008; Goubault et al., 2019). Unsurprisingly, an increase in bradykinesia raised the risk to be affected in fine motor tasks, but the model suggests that this is true only for the subset of the sample also affected in mobility tasks. Indeed, bradykinesia by itself did not come out as a significant factor to discriminate participants with normal performance and participants slightly affected in fine motor tasks (i.e., clusters 1 and 2). However, an increase in residual (i.e., on medication) bradykinesia increased the likelihood of being affected in all domains as opposed to being affected only in mobility tasks, suggesting that this factor is more relevant to appendicular rather than axial motor control. Only dyskinesia came out as a significant factor in the differentiation between patients with normal performance and patients slightly affected in fine motor tasks. Indeed, the present way of analyzing this data confirms what has been described in previous studies (Goubault et al., 2019) using the same patients that dyskinesia increases, to a certain extent, the likelihood of being in the *normal* performance group when compared to the group slightly affected in fine motor tasks or the group affected in mobility. We acknowledge the fact that few patients displayed severe dyskinesia in the current sample, but low to moderate levels of dyskinesia certainly did not interfere with the patients' performance. Results have also demonstrated that when all other symptoms are equivalent, the addition of Amantadine in the medication regimen decreases the risk of being part of the cluster affected in mobility task, when compared to the normal performance group. These results are concordant with the effect of Amantadine on gait in PD patients under deep-brain stimulation [16]. Yet, the impact of Amantadine on gait is still unclear [17], as well as the fraction of benefit that may derive from the reduction in levodopa daily dose afforded by this drug. Cognitive impairment did not come out as a global significant factor, but it did have a significant impact in differencing people with disabled mobility.

It is worth mentioning that the reported results could have been different if patients were tested in their OFF state. Indeed, all patients were tested at peak dose, assuming medication was optimal. The reported impact of the different symptoms on the performance cluster affiliation therefore refers to the effect of the residual symptoms. Further studies should consider running similar analyses ON and OFF states to assess not only the direct impact of the symptoms, but also to bring one step further the analysis of the medication's impact instead of only considering the number of years since diagnosis in the analyses. Another limit to the current study regards the subset of tasks used for the analysis. Future work will focus on applying a similar protocol on the entire set of tasks collected.

The statistical model developed using the global patient symptomatology allowed to predict the impact of the disease on the patients' motor repertoire with an accuracy of 76%. The model was specifically good at recognizing patients with mobility and global issues (i.e., clusters 3 and 4). Such results demonstrate the strength of the global approach, although future work should investigate other classification approaches to improve the overall accuracy. For example, machine learning approaches with a K-fold cross-validation loop could improve the accuracy of the classification process. The general approach also needs to be tested on a much larger group of patients and by using traditional clinical testing to render it more usable. We could then be able to determine, based on that evaluation, what will be the impact of the symptomatology of the patient's ADL, and as such predict their ability to perform everyday tasks.

## Conclusion

PD affects the motor repertoire of patients to different extents. This study demonstrated that four major performance profiles appear to exist: patients with normal performance, patients affected slightly in fine motor tasks, patients affected in mobility tasks and patients affected in all domains of mobility. This study demonstrated that it is possible to predict the mobility performance of any patient, based on personal clinical features. Although future research is needed to refine the clustering method, as well as performance prediction suiting clinical evaluations, these results appear promising, and may lead to more personalized treatment by identifying and targeting symptoms that specifically impede a particular patient's motor performance.

## Data Availability Statement

The raw data supporting the conclusions of this article will be made available by the authors, without undue reservation, to any qualified researcher.

## Ethics Statement

The studies involving human participants were reviewed and approved by the Comité d'éthique de la recherche vieillissement-neuroimagerie du CIUSSS du Centre-Sud-de-l'l^le-de-Montréal. The patients/participants provided their written informed consent to participate in this study.

## Author Contributions

KL developed the algorithms, designed the analysis, and drafted the manuscript. EG and SB collected the data and provided significant feedback on the study analysis and the paper. CD conceived the experiment, helped in data interpretation, and reviewed the paper. PB was significantly involved in the interpretation of the data and the review of the manuscript.

### Conflict of Interest

The authors declare that the research was conducted in the absence of any commercial or financial relationships that could be construed as a potential conflict of interest.
